# The Energy of COPI for Budding Membranes

**DOI:** 10.1371/journal.pone.0133757

**Published:** 2015-07-28

**Authors:** Abdou Rachid Thiam, Frédéric Pincet

**Affiliations:** 1 Laboratoire de Physique Statistique, Ecole Normale Supérieure de Paris, Université Pierre et Marie Curie, Université Paris Diderot, Centre National de la Recherche Scientifique, 24 rue Lhomond, 75005, Paris, France; 2 Department of Cell Biology, School of Medicine, Yale University, 333 Cedar Street, New Haven, CT 06520, United States of America; Institut Curie, FRANCE

## Abstract

As a major actor of cellular trafficking, COPI coat proteins assemble on membranes and locally bend them to bud 60 nm-size coated particles. Budding requires the energy of the coat assembly to overcome the one necessary to deform the membrane which primarily depends on the bending modulus and surface tension, γ. Using a COPI-induced oil nanodroplet formation approach, we modulated the budding of nanodroplets using various amounts and types of surfactant. We found a Heaviside-like dependence between the budding efficiency and γ: budding was only dependent on γ and occurred beneath 1.3 mN/m. With the sole contribution of γ to the membrane deformation energy, we assessed that COPI supplies ~1500 k_B_T for budding particles from membranes, which is consistent with common membrane deformation energies. Our results highlight how a simple remodeling of the composition of membranes could mechanically modulate budding in cells.

## Introduction

Coat proteins, namely Clathrin coats, coat protein complex I (COPI) and II (COPII) perform a critical step of intracellular vesicle trafficking. They respectively form vesicles from the plasma, the Golgi and the endoplasmic reticulum membranes, exhibiting different morphology and mechanical properties. To induce vesicle formation, monomers of the coat protein machineries, called coatomers, assemble on the target membrane and polymerize to locally bud nanometer sized spherical caged-particles of given curvature [1, 2]. This budding process is biochemically and mechanically regulated [3, 4]. Biochemical regulation is inherent to local variation of one or several components of the coat protein machineries [4]. Mechanical regulation occurs by variations of the bending modulus κ, e.g. by remodeling of membrane composition, and the surface tension γ, e.g. by changing the membrane surfactant density [5, 6]. These mechanical parameters define the minimal energy for budding off a particle of radius r, E = 8πκ+4πγr^2^, the sum of the bending and stretching energies. This minimal energy which is presumably different for each organelle membrane has to be met by the polymerization energy of the coatomers, E*, to form spherical coats enclosing the particles. Knowing E* for each coat protein machinery will bring important and new knowledge on biochemical and biophysical regulation of cellular trafficking. Previous theoretical attempts based on the comparison between the bending energy of bilayers and the elasticity of dilation of bilayer-bound coat proteins [7, 8] suggest E* to be of the order of 2000 k_B_T.

Of the three coat proteins, only COPI was shown to act *in vivo* on both phospholipid bilayers and monolayers, namely on the Golgi apparatus and lipid droplets which are organelles at the core of cellular energy metabolism [4, 9–11]. Because the Golgi has a very low surface tension γ (<<1 mN/m) [12], deforming its membrane is almost solely dependent on the bending modulus[13, 14] κ, ~20 k_B_T, whose contribution to E is predominant. In contrast to the Golgi, lipid droplets are covered by a single phospholipid monolayer membrane. The surface tension of this type of membrane was determined for triolein emulsion droplets to be between 1 to 40 mN/m [5, 15], much higher than that of the Golgi bilayer. Hence, for lipid droplets, the contribution of γ becomes very important for the membrane deformation energy [5, 16].

The ability of COPI to bud nanoparticles from a monolayer or bilayer membrane can be predicted knowing E*^COPI^, the energy supplied by the polymerization of COPI coatomers. Measuring E*^COPI^ in cells is experimentally challenging because the mechanical parameters are not controlled, membranes are dynamic systems and other proteins may interfere with them, and finally visualization of the coat formation is difficult. So far, various *in vitro* approaches, based on unilamellar vesicles [14, 17–19], or cell membrane extracts [20, 21], were used to exclusively study the ability of coat proteins to form vesicles. These approaches probed the biochemical triggering of budding and well described the molecular details of coatomer assembly mechanisms. The description of the energy landscape of the budding process is however still lacking because of the challenge to concomitantly visualize budded coat-vesicles with controlled membrane parameters.

Using a recently developed COPI-induced oil nanodroplet formation approach [5], we worked with different amounts and types of surfactant in the oil, to vary membrane mechanical properties, and studied how they influence nanodroplets budding. We found that the efficiency of the budding reaction depends on the surfactant type. However, a direct Heaviside-like dependence between the budding efficiency and γ was found, independently of the surfactant. Budding was mainly opposed by γ and occurred only beneath 1.3 mN/m. This simple dependency upon γ was expected for the emulsion monolayer membrane in our experiment because it was presumed that other mechanical terms have a minor contribution to budding. Hence, we used the sole contribution of the stretching energy due to γ to determine that COPI supplies an energy of ~1500 k_B_T to bud membranes.

## Materials and Methods

### Preparation of the solutions

Phospholipids (PLs) and the triolein solution: we chose a lipid composition close to that of cellular natural lipid droplets [5]: Dioleoylphosphatidylcholine, Dioleoylphosphatidylethanolamine, Cholesterol, Lyso-Phosphatidylinositol, Lyso-Phosphatidylethanolamine, Lyso-Phosphatidylcholine (50:20:12:10:5:3). All phospholipids were purchased from Avanti Polar Lipids. The oil phase consisted of triolein (TO) purchased from Sigma Aldrich. The phospholipids were initially solubilized in chloroform that was first evaporated under vacuum. We then added the required amount of TO to the PLs to reach the desired concentration. The mixture was then vortex mixed for 2 min and subsequently sonicated for 1 min.

Cosurfactants (COs), Oleic acid (OA) and dioleoyl glycerol (DOG), were purchased from Sigma Aldrich.

### Generation of the buffer drops

**Fig 1:**
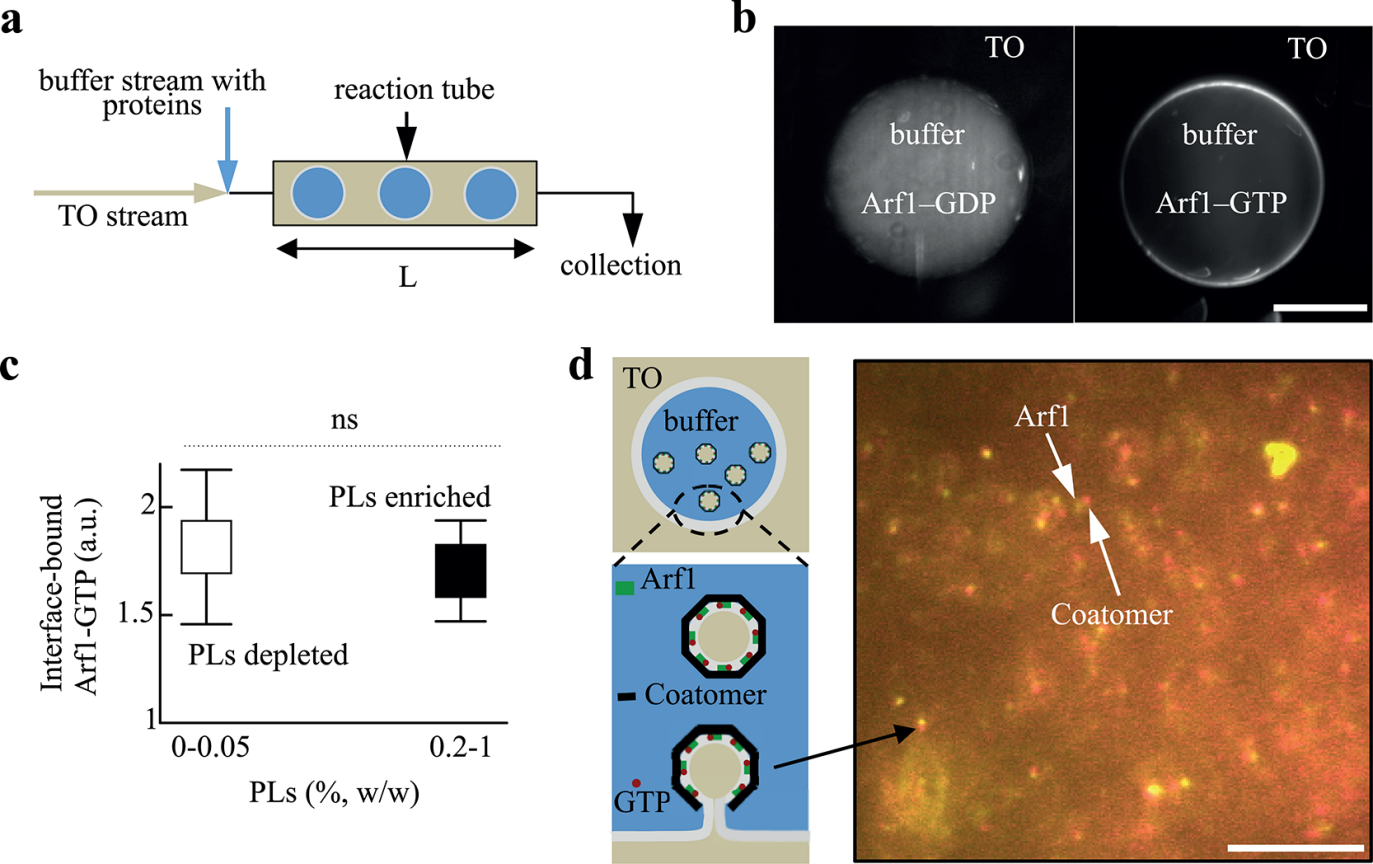
(a) Buffer drops formation. Triolein oil (TO) and buffer solutions were flown into a t-shape connector attached to a Teflon tube. Buffer drops encapsulating the proteins were formed in TO, inside the tube; they were collected from the tip of the tube for quantification. (b) Buffer drops containing Arf1-Cy3 (100 nM), in GTP (10 mM) or GDP bound form, and Arno (100 nM), were separately formed, arrested in the tube, and visualized by fluorescence. Arf1-GTP bound the interface (right panel); Arf1-GDP remained in volume (left). Scale bar is 100 μm. (c) Changing the amount of phospholipid (PL) did not impact the recruitment of Arf1-GTP to the interface. The ratios of Arf1-Cy3 signal between the membrane and the volume, with or without PLs (depleted, 0% PLs, enriched, 0.2% PLs (w/w to TO)) were similar; at 0.05% and 1%, the same trend was observed; about 20 drops were quantified for each case; the p-value is 0.3 and ns stands for non-specific. (d) Budding scheme of Arf1/COPI from the buffer/TO interface and visualization of a buffer microreactor content. The presence of colocalized Arf1-Cy3 (100 nM) and coatomer-Alexa647 (25 nM) spots reveals budding activity at the buffer interface as described in the scheme. Homogeneous particles (60 nm TO droplets) were budded inside the buffer drop as described in [5] and S1 Video. The frequent drift occurring between red and green signals is due to a time delay required for switching the lasers while particles were in motion in solution. Scale bar is 10μm.

The TO lipid oil and the buffer phase (50 mM Hepes, 120 mM Kacetate, and 1 mM MgCl_2_ in MilliQ water) containing the proteins were stored into different syringes serving for injecting them in a T-connector attached to a Teflon tube (Fig 1A). Buffer drops were thus generated into the oil phase and arrested in the tube; they were then observed by fluorescence (Fig 1B–1D). When budding occurred, each buffer drop contained COPI-TO nanodroplets, which were collected at the tip of the Teflon tube and analyzed (Fig 1A and [5]).

### Quantification of the budding process

**Fig 2:**
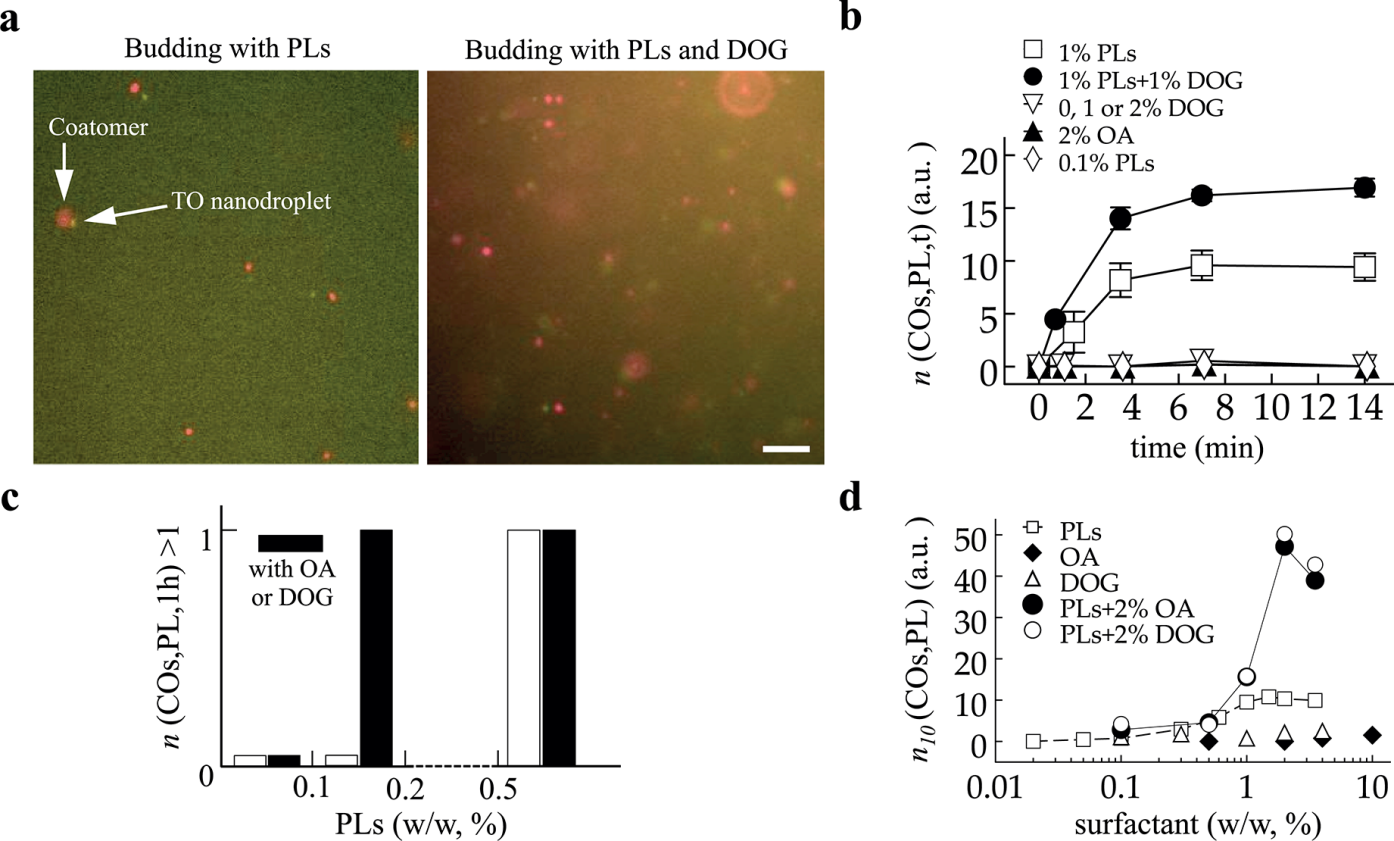
(a) Visualization of budded nanodroplets collected from microreactors after 10 min of reaction time. Coatomer and oil signals were recorded. Two cases of low and high COPI budding efficiency respectively due to the presence of low and high PL content with DOG (2%, w/w to TO) in TO. Scale bar is 10μm. (b) Evolution of the number of budded nanodroplets over time. Microreactors were collected at different time points and the number of budded nanodroplets per area *n*(COs,PL,t) was determined for various membrane conditions (e.g. 1% PLs or 2% DOG). Different plateaus were obtained for the two budding cases. (c) Occurrence of budding after one hour to visualize the non-budding/budding transition. When no budding was observed, the bar was set at zero; when *n*(COs,PL,1h) > 1 the bar was set at 1, independently of the value of *n*(COs,PL,1h). PL alone is represented in white bars. At 0.2% PLs and below, no budding occurred; at 0.5% PL and above budding occurred. Black bars correspond to the presence of COs in TO. Budding occurred only above 0.2%PL with 2% COs. The sole presence of 0, 1, 2, 10% of COs did not mediate budding at all after one hour reaction time. (d) Budding quantification after 10 min reaction time, *n*_*10*_(COs,PL), for different formulations. The conditions are TO + 0 or 2% COs with various amounts of PLs, or TO in which the concentration COs was varied; the term surfactant refers to either PL or COs variation. COs alone did not allow budding. However, 2% COs increased the number of budded nanodroplets in presence of PLs.

Budded particles were collected after the budding reaction at the tip of the tube (Fig 1A). The collected sample was placed in a chamber whose bottom was made of glass, coated with a thin polydimethylsiloxane polymer layer (10 μm) to prevent sticking of the particles. Imaging of the particles was done using an inverted epifluorescence Zeiss microscope equipped with a 63x oil objective. To identify the budded particles, two emission channels were simultaneously recorded: TO labeled with Bodipy-green (from Lifetechnologies) and COPI (Alexa-647). The nanodroplets were identified as tiny particles having both markers. The budding process was quantified following our previous work [5]. For each probed membrane condition, we focused in the volume of the sample (focal depth 4 μm, field of view of 130 μm x 130 μm), took snapshots every minute (ten times) and counted the number of protein-coated nanodroplets (e.g. Fig 2A); we changed the focal plan at least twice for each experiment. Finally, only fluorescence intensities of coatomer spots between 20–100% of the maximum coatomer signal in the focal plan were counted, as they corresponded to the most visible spots in the focal volume [5]; the laser power was kept constant.

We averaged the counted number of budded particles per area (130 μm x 130 μm) and determined their standard deviation. This number of budded particles per area is *a priori* dependent on the composition of the monolayer, cosurfactants (COs), phospholipids (PLs), and time, t; in the text, it is denoted *n*(COs,PL,t).

### Surface tension measurements

Surface tension was measured using the drop weight method [22] and micropipette aspiration technique [5, 23]. These methods are described in our previous work [5].

Briefly, in the drop weight method, a buffer drop was continuously and slowly flown, at a flow rate of 20 μl/hr to allow dynamic interfacial equilibrium, in a TO oil phase containing a given concentration of surfactant. At a critical size it detaches. For each concentration, an image of the drop was taken every 5 sec. From the inner diameter d of the injection tube (d = 250 μm), the surface tension was given by m*g/(π*d*f) where f is a Wilkinson geometric parameter correction that depends on the ratio between d and the radius of the detached drop; g is the gravity constant. The mass m of the drop was calculated according to m = vΔρ (v is the drop volume and Δρ is the density difference between oil and buffer). Surface tension values measured by this method were in accordance with those obtained by the micropipette technique.

The device of the micropipette technique consisted of a micromanipulator and a pipette holder (Narishige). Pipettes were incubated in a 5% (wt/vol) bovine serum albumin solution before use, so as to prevent the adhesion of the droplets to the glass. Micromanipulation of a single droplet enabled determining the interfacial tension (γ) through measurement of the pipette radius, R_p_, droplet radius, R_d_, and the minimal aspiration pressure at which the droplet was drawn into the pipette, P_suc_, following the equation:

$$\gamma ={\text{P}}_{\text{suc}}/\left(2\left(1/\;{\text{R}}_{\text{p}}\text{-}1/\;{\text{R}}_{\text{d}}\right)\right).$$

The suction of the droplet was carried out using a syringe. The resulting pressure was measured with a pressure transducer (DP103; Validyne Engineering Corp.), the output voltage being monitored with a digital voltmeter. The pressure transducer (in the range of 55 kPa) was calibrated before experiments.

## Results and Discussion

We recently developed an assay in which the COPI machinery acts on the monolayer of a buffer/TO interface, mimicking the surface of lipid droplets, to bud 60 nm-COPI-coated TO oil nanodroplets [5, 9]. In a basic microfluidic device, 200 μm-size buffer drops, encapsulating the COPI machinery, were generated in an environment consisting of a TO oil phase containing controlled amounts of phospholipids and/or other lipid surfactants (Fig 1A). As soon as the buffer microreactors were formed in the oil phase, a surfactant monolayer was bound to their interface. The properties of the monolayer remained constant during the experiments as the surrounding TO phase represents a reservoir of surfactants. We were able to show that the encapsulated COPI machinery uses this surfactant monolayer as a substrate to generate the 60 nm-COPI-coated TO particles (Fig 1D and S1 Video) which were confirmed by electron microscopy, fluorescence imaging, and fluorescence correlation spectroscopy [5].

To initiate the action of COPI machinery on a bilayer membrane, a small GTPase, ADP-ribosylation factor 1, Arf1, first strongly binds to the membrane, in presence of GTP and an exchange factor, e.g. Arno. For comparison, we observed that Arf1 also binds the buffer/TO monolayer interface in a GTP-specific manner (Fig 1B). The binding of the protein was moreover independent of the membrane composition (Fig 1C), as we varied over a wide range the surfactant concentration in the oil and observed no significant change in the amount of recruited protein. Based on this result, we considered that the contribution of Arf1 into the budding process was independent of membrane composition. Bound-Arf1 recruits coatomers that polymerize into a 60 nm-spherical cap enclosing TO particles ([5] and S1 Video). Since Arf1 recruitment did not depend on membrane composition, variation of the number of budded nanodroplets was exclusively assigned to the ability for COPI coatomers to fully polymerize in a spherical cap. Release of the budded particles in the microreactors requires the fission of the buds from the interface. Our study provides readout for both budding and fission together but can not discriminate between them. In the following, we will simply refer to nanodroplets formation, i.e. budding and fission, by budding.

### The number of budded particles increases and saturates over time

We worked at 7 nM coatomer-Alexa647 and 100 nM Arf1 in the buffer microreactors, to make sure that, for each probed membrane condition, the kinetics of the proteins recruitment was not limiting, and that many nanodroplets will be formed under budding conditions. We generated buffer microreactors that were incubated for different times in the TO phase before collecting them. To achieve this, flow rates were kept constant (respectively at 1200 and 100 μl/h) and the length L of the reaction tube was varied (Fig 1A). In the TO oil phase, we varied the composition of the monolayer; it contained different concentrations of oleic acid (OA), dioleoylglycerol (DOG), phospholipids (PLs), or their combination. We did not see differences between OA and DOG in our experiments; when referring to both of them in the text we will use the annotation COs.

For each monolayer composition leading to budding, e.g. 1% PL (Fig 2B), the number of budded nanodroplets per area over time *n*(COs,PL,t) (see Materials and Methods and [5]) displayed a monotonic increase before reaching a plateau after 6 mins. The plateau or saturation of *n*(COs,PL,t) was expected because the concentration of free coatomers, which were consumed during budding, gradually decreased over time. For non-budding compositions, *n*(COs,PL,t) was zero even after 14 min reaction time, e.g. for 2% DOG or 0.1% PLs. We probed whether under these conditions budding could occur at longer time points. After 1h, having for example 1%, 2%, or 10% of COs in TO and/or less than 0.1% PLs, did not allow budding (Fig 2C); increasing the protein concentration by ten folds did not promote budding either after one hour. These results suggest that certain monolayer compositions were not favorable for budding, regardless of COPI concentration or how long the proteins were in contact with the monolayer. Conversely, budding was always observed in the presence of PLs at concentrations over 0.3% (Fig 2C).

Under budding conditions, we also observed different saturation levels of *n*(COs,PL,t), which were higher in the presence of 2% of COs for example (Fig 2A and 2B). In reality, COPI coatomers get inactivated after several minutes in solution, as they are formed by seven complexes that disassemble over time. This is the reason why we sought to work at high protein concentration to allow visualization of budding events despite disassembly and coatomer inactivation. The inactivation of the protein contributed of course to the saturation of budding but was essentially the reason for the difference observed between the saturation levels observed in Fig 2B and visible in Fig 2D. Without the inactivation, we would expect both cases to reach the same plateau but at different time points.

In short, different parameters such as membrane composition for budding conditions and coatomer polymerization or disassembly affected the number of budded nanodroplets (Fig 2A and 2B). However, only membrane composition was responsible for the non-occurrence of budding, as increasing the concentration of proteins or reaction time did not promote budding (Fig 2C). Our results thus strongly suggest that regardless of time membrane composition was the key parameter determining budding occurrence, even though the number of formed nanodroplets may vary. This conclusion is valid as long as the reaction time is larger than the time scale of the full assembly of coatomers, which was of the order of a 1 minute as inferred from Fig 2B.

We conclude the existence of a budding transition dependent on membrane physical parameters. To investigate this transition, we varied the surfactant composition and concentration and determined *n*_*10*_(COs,PL), the number of formed nanodroplets per area after 10 min of reaction time, as there were more particles to count. We determined *n*_*10*_(COs,0%), *n*_*10*_(0%,PL), *n*_*10*_(2%,PL) and observed that having exclusively COs did not lead to particle formation by COPI (Fig 2D), regardless of their concentration, varied up to 10%, i.e. *n*_*10*_(COs,0%) ~ 0. As already shown [5], *n*_*10*_(0%,PL) increased continuously up to 1% PL where it reached a maximum. Having additional 2% of OA or DOG as cosurfactants in TO increased *n*_*10*_(2%,PL) compared to *n*_*10*_(0%,PL). For example, by keeping the total surfactant amount at 4% in TO *n*_*10*_(2%,2%) was five-fold larger than *n*_*10*_(0%,4%) (Fig 2D). To understand these differences, mainly the effect of COs, we focused on COs = 0% and COs = 2% and used the normalized number of nanodroplets as *B*_*0*_(PL) = *n*_*10*_(0%,PL)/max(*n*_*10*_(0%,PL)) and *B*_*2*_(PL) = *n*_*10*_(2%,PL)/max(*n*_*10*_(2%,PL)). *B*_*COs*_ is defined as the budding efficiency for each COs concentration.

### COPI budding efficiency is controlled by surface tension

**Fig 3:**
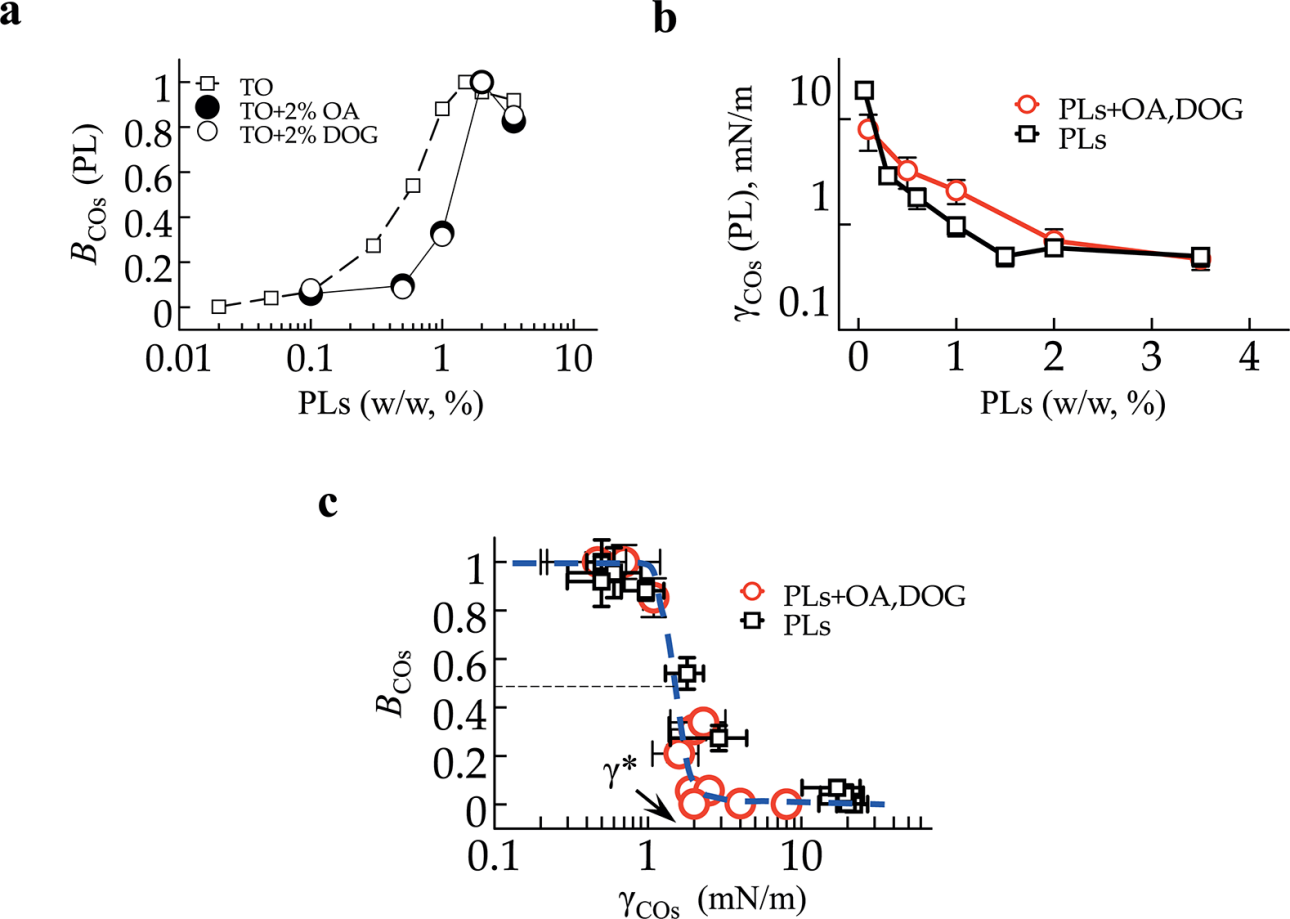
(a) For each budding formulation in Fig 2D, i.e. TO or TO + 2% COs with variable PL concentration, the budding efficiency *B*_COs_ = *n*_*10*_/max(*n*_*10*_) was plotted. The transition of budding is shifted to higher PL concentrations in presence of COs. (b) The surface tension γ for each condition of (a) was measured; it exhibits an opposite variation to γ_PL_, as there is a switch between circle and square symbols between (a) and (b). (c) The budding efficiency *B*_COs_ in logarithmic dependence of γ_COs_. All data points follow a Heaviside-like master curve. The critical tension for budding is γ = 1.3 ± 0.2 mN/m.

We represented *B*_*0*_ and *B*_*2*_ (Fig 3A) against the PL concentration. We observed a shift of the budding efficiency profile of *B*_*0*_(PL) towards higher phospholipid concentrations to obtain *B*_*2*_(PL).

For the monolayer membrane of emulsion droplets, the surface tension γ is the relevant energy parameter of the membrane that may oppose the budding of nanodroplets. We measured γ_COs_(PL) (Fig 3B) and observed that it evolved conversely to B_COs_, and the shift observed in the presence COs was conserved (compare the position of circles and squares in Fig 3A and 3B). The surface tension increase of the PL monolayer owing to the presence of OA and DOG can be unexpected. In fact, OA and DOG are highly soluble in the TO oil phase, much more than PLs. Although they act as surfactants, they solubilize part of the PLs from the buffer/TO interface back into the oil, e.g. into micelles, thereby increasing surface tension, and vice versa. This probably explains the higher number of *n*_*10*_(2%,2%) in Fig 2D compared to *n*_*10*_(0%,4%). Indeed, during budding, the PL monolayer of the microreactors is consumed by nanodroplets; for budding to continue, PLs in the TO have to quickly replenish the microreactors monolayer, which could have been facilitated by OA and DOG.

We represented *B*_COs_ against γ_COs_ and observed that all experimental data points follow a Heaviside-like master curve (Fig 3C) regardless of membrane composition. This supports our results (Fig 2B and 2C) that budding is either complete or never occurs for a given membrane composition in a time scale less than one hour. The budding efficiency consequently depended on the sole surface tension. Consistent with this finding, the exclusive modulation of vesicle budding by γ from giant unilamellar vesicles was shown [17].

### The energy of COPI polymerization is ~1500–2000 k_B_T

The required energy for nanodroplet formation can be written as the sum of bending and stretching energies, E = 8πκ+4πγr^2^. For emulsion droplets with fluid monolayer membranes, e.g. at the buffer/TO interface, the value of κ is generally beneath 10 k_B_T [15, 24, 25]. In our experimental system, the contribution of 8πκ to the deformation energy would be thus constant and low, beneath 250 k_B_T, while the contribution of the γ term was at least 1000 k_B_T. The predominance of the stretching energy in our system explains the Heaviside-like dependence of budding with γ (Fig 3C) and offers a unique and simple way to assess E*^COPI^. Continuously varying γ, by almost a hundred fold (Fig 3B and 3C), was de facto tuning the resistance of the membrane to deformation. Because budding occurred predominantly below 1 mN/m and was inexistent above 2 mN/m, with a sharp transition in between, we inferred the critical surface tension value, γ*, beneath which COPI can form nanodroplets. The value of γ* corresponds to the mid-point of the transition indicated in Fig 3C, corresponding to γ* = 1.3±0.2 mN/m.

At the value of γ*, E*^COPI^ and the nanodroplet formation energy, 4πγ*r^2^+8πκ are exactly balanced. The 60 nm-size budded nanodroplets have a 10 nm-thick coat [5, 18] and consequently an actual diameter of 2r = 40 nm. The stretching energy (4πγ*r^2^) was therefore ~1500 k_B_T. By taking into account the bending energy (8πκ) which was a priori constant and lower than 250 k_B_T, we found E*^COPI^ ~1500–2000 k_B_T, in good agreement with previous theoretical predictions of less than 2000 k_B_T [7, 8]. This value also suggests that in cells the largest bending modulus of a membrane which COPI can fully bud is κ* = E*^COPI^/8π ~ 60 k_B_T.

### Potential mechanical and biochemical regulations of budding in cells

In a cellular context, the contributions of κ and γ evolve complexly due to frequent membrane remodeling. Mechanical regulation of budding by coat proteins means that membrane properties are dynamically adapted by cell activity between budding and non-budding physical states, based on E*^COPI^.

For a monolayer membrane such as of lipid droplets, γ will essentially regulate the ability of COPI to perform budding, as a substantial increase of κ is unlikely [25]. The budding of COPI particles will for example quickly increase the surface tension of the monolayer, which will in return decrease and arrest the process when its value will be beyond γ*. For the Golgi bilayer membrane, COPI mechanical regulation is more complex, as both contributions of κ and γ can be of similar importance. However, regulation by γ can be inferred from the occurrence of budding or fusion events of vesicles from/to a bilayer [14, 17], respectively inducing an increase or a decrease of the bilayer tension. This was well illustrated by the reconstitution of COPI vesicle budding from an initially deflated giant unilamellar vesicle (GUV) [17]. The budding of many vesicles resulted to an increase of the GUV surface tension, which arrested the process [17]. This control of budding by surface tension was well illustrated in cells by the gradual inhibition of COPI vesicle formation from swelling Golgi [26].

The value of E*^COPI^ also gives insights on the molecular dynamics of the coat proteins and their biochemical regulation. Forming a 60 nm-COPI coated particle involves n = 50 to 70 coatomers [27]. Each coatomer binds to 1 to 4 Arf1 proteins [4] and to 3 other coatomers [27]. The energy per bound-coatomer arising from the binding to Arf1 and to neighboring coatomers is E*^COPI^/n ~ 20–30 k_B_T. This value is in good agreement with the prediction that vesicle budding should occur only if coatomers interaction overcomes κ~20 k_B_T [7, 8], the bilayer bending modulus. Assuming the binding to Arf1 do not have substantial contributions to budding, coatomer dimerization supplies an energy e_d_~10 k_B_T corresponding to a lifetime τ = τ_0_exp (e_d_/k_B_T) (with τ_0_<10^-8^s [28]) shorter than ~1 ms. With respect to organelles, this result shows that a COPI coat network will nucleate and grow only if several cascade of coatomer bonds are formed in less than 1 ms. The bond between two coatomers must be rapidly stabilized to prevent their disassembly. Stabilization of the forming spherical cap can be achieved by the rapid binding to other coatomers which is favored by increasing the coatomer concentration. In contrast, lowering the local COPI concentration, below a threshold at which the rate of coat bonds formation is slower than a few bonds per ms, will substantially prevent the coat growth [29]. Interaction of Arf1/coatomers with cargo receptors can also contribute to stabilize the emerging coat [4].

## Conclusion

The emulsion-based approach presented here allowed us determining the COPI energy for budding membranes. Knowing this energy, which is ~1500–2000 k_B_T, offers good projections on the mechanical and biochemical features of COPI coat assembly. Establishing the energies for other coat proteins would be of great interest to understand the difference and relevance between the molecular topologies of the proteins. These comparisons can be generalized to other proteins inducing membrane deformation such as Caveolins or BAR proteins. Knowing their energy value will bring considerable knowledge on the biological importance of membrane remodeling during cellular trafficking.

## Supporting Information

S1 Video Movie of the recovered budded oil nanodroplets. The product of the microreactors were recovered and placed between two cover slips. The oil signal is only shown. The nanodroplets are homogeneous. The number of nanodroplets decreased over time due to bleaching. The size of the field is 60 μm.
(AVI)
